# Twelve Positions in a β-Lactamase That Can Expand Its Substrate Spectrum with a Single Amino Acid Substitution

**DOI:** 10.1371/journal.pone.0037585

**Published:** 2012-05-22

**Authors:** Hyojeong Yi, Kwang-Hwi Cho, Yun Sung Cho, Karan Kim, William C. Nierman, Heenam Stanley Kim

**Affiliations:** 1 Department of Medicine, College of Medicine, Korea University, Seoul, Korea; 2 School of Systems Biomedical Science and Research Center for Integrative Basic Science, Soongsil University, Seoul, Korea; 3 J. Craig Venter Institute, Rockville, Maryland, United States of America; University of Canterbury, New Zealand

## Abstract

The continuous evolution of β-lactamases resulting in bacterial resistance to β-lactam antibiotics is a major concern in public health, and yet the underlying molecular basis or the pattern of such evolution is largely unknown. We investigated the mechanics of the substrate fspectrum expansion of the class A β-lactamase using PenA of *Burkholderia thailandensis* as a model. By analyzing 516 mutated enzymes that acquired the ceftazidime-hydrolyzing activity, we found twelve positions with single amino acid substitutions (altogether twenty-nine different substitutions), co-localized at the active-site pocket area. The ceftazidime MIC (minimum inhibitory concentration) levels and the relative frequency in the occurrence of substitutions did not correlate well with each other, and the latter appeared be largely influenced by the intrinsic mutational biases present in bacteria. Simulation studies suggested that all substitutions caused a congruent effect, expanding the space in a conserved structure called the omega loop, which in turn increased flexibility at the active site. A second phase of selection, in which the mutants were placed under increased antibiotic pressure, did not result in a second mutation in the coding region, but a mutation that increased gene expression arose in the promoter. This result suggests that the twelve amino acid positions and their specific substitutions in PenA may represent a comprehensive repertoire of the enzyme’s adaptability to a new substrate. These mapped substitutions represent a comprehensive set of general mechanical paths to substrate spectrum expansion in class A β-lactamases that all share a functional evolutionary mechanism using common conserved residues.

## Introduction

Enzymatic hydrolysis has been the most common mode of bacterial resistance to β-lactam variants, as β-lactamases are apt to expand their substrate spectrum with simple mutations [1], [2], [3]. Since the identification of the first extended-spectrum β-lactamases (ESBLs) in the 1980s, members of the ESBLs have been steadily increasing following the release of new man-made drugs [4], [5], [6]. Analyses of amino acid substitutions in these enzymes have enhanced our current understanding of substrate spectrum expansion in class A β-lactamases [1], [4]. However, much of the molecular basis that underlies the adaptability of these enzymes to new antibiotics remains to be understood. Even in the most extensively studied TEM-type ESBLs, only a small number of amino acid substitutions have been confirmed to have a direct role in the enzyme’s expanded substrate spectrum: ten amino acid substitutions against β-lactam antibiotics, six against β-lactamase inhibitors, and two against both [4].

To profile the adaptability of class A β-lactamases to a new antibiotic, we conducted a large-scale mutant selection with the chromosomally encoded PenA from *B. thailandensis* [*7*] (BTH_II1450 from *B. thailandensis* strain E264) to investigate its adaptation to an oxy-imino-cephalosporin, ceftazidime. *B. thailandensis* is resistant to penicillins [8], and therefore is a good system for a substrate expansion study of this kind. Furthermore, because *B. thailandensis* is a non-pathogenic soil saprophyte [7], it is unlikely that this bacterium has been heavily exposed to ceftazidime. We expected that with *penA* we would be able to observe fresh mutational responses to the challenges posed by exposure to the new ceftazidime drug. Furthermore, *penA* is a closely related ortholog to genes in the pathogenic species *B. pseudomallei, B. mallei,* and the *Burkholderia cepacia* complex (Bcc) [9], [10], [11], and the antibiotic regimen used to treat infections by these bacteria generally includes ceftazidime [12], [13].

*B. pseudomallei* is the etiological agent of septicemic melioidosis, which is endemic in Southeast Asia and Northeastern Australia [14]. *B. mallei*, the cause of glanders, is a species derived from a clone of *B. pseudomallei* [15]. The Bcc, which is a complex composed of more than 10 *Burkholderia* species, including *B. cepacia*, *B. cenocepacia*, and *B. multivorans*, is a group of nosocomial pathogens of increasing concern as these bacteria cause respiratory and systemic infections in patients with cystic fibrosis (CF) or chronic granulomatous disease, and in other immuno-compromised patients [12]. To date, only a few cases of ceftazidime resistance in *B. pseudomallei* and Bcc have been reported and resistance in most of these cases pointed to a single gene [9], [10], [11], [16]. Two single amino acid substitutions, Pro167Ser and Cys69Tyr (amino acid residue numbering following Ambler *et al.* [17]), have been described in PenA of ceftazidime resistant *B. pseudomallei* isolates [10], [11], [16]. Similarly, two orthologs of PenA, PenB2 and PenB3, with 7 and 2 amino acid alterations, respectively, were shown to be associated with ceftazidime resistance in clinical isolates of *B. cenocepacia* [9].

## Results and Discussion

### All 516 Ceftazidime-resistant Mutants had a Mutation in the *penA* Gene

We measured the intrinsic level of the susceptibility of the wild-type *B. thailandensis* to ceftazidime to determine the appropriate antibiotic pressure for the experiment (see Materials and Methods). Then, a concentration (5 µg/ml) slightly lower than three times the wild-type MIC value (1.75 µg/ml based on the E-TEST, Table S1) was used in the mutant selection process. From a total of 1×10^8^ cells on a selection plate, 1 to 10 cells survived to form visible colonies after 16–20 h incubation, resulting in an estimated mutation frequency of 10^−8^ to 10^−7^.

**Figure 1:**
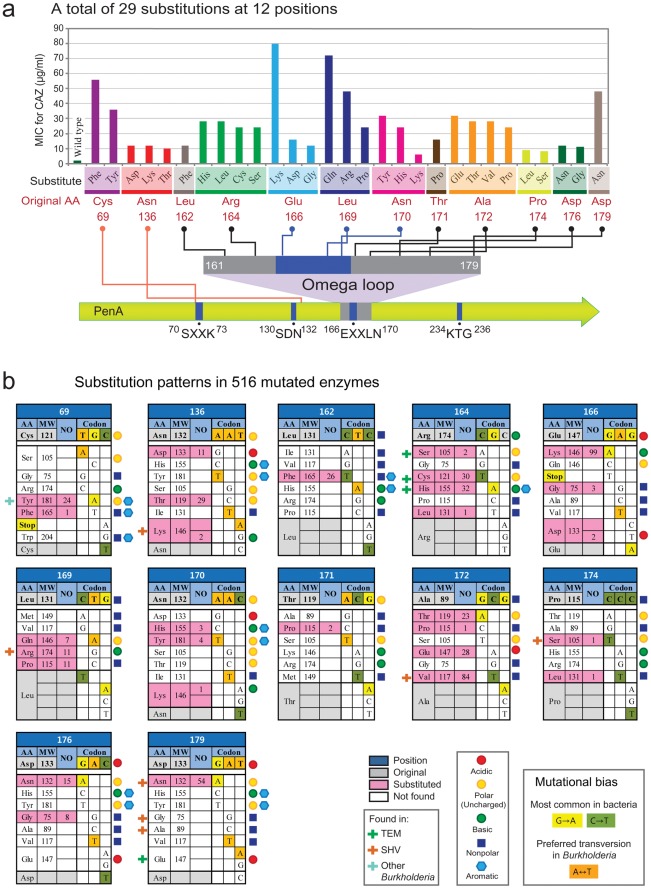
Single amino acid substitutions in PenA. a. Map of the twelve positions and twenty nine amino acid substitutions. The PenA protein is represented by an arrow, on which four conserved domains of sequences and the omega loop are indicated. The twelve positions are denoted by position numbers (numbering according to Ambler *et al*. [17]) and the amino acid residues are denoted using the three-letter code. Amino acid substitutions are shown above the original amino acids. The MIC levels for ceftazidime are shown in the bar graph. b. Substitution patterns in 516 mutated PenA enzymes. For each position, the amino acid residue (AA) of the original and those coded for based on a possible single-nucleotide mutation are listed with molecular weight (MW) and the number of occurrence (NO). Substitutions previously found in other class A β-lactamases, that are based on the data from the Lahey Clinic (http://www.lahey.org/Studies/), are denoted by another set of colored symbols.

To investigate the mutation patterns of *penA*, we sequenced the gene in resistant *B. thailandensis* isolates. Intriguingly, all 516 isolates contained a single point mutation in the coding region of *penA* (Fig. 1a). To confirm that *penA* genes with a mutation (hereafter referred to as penA*s) were responsible for the ceftazidime-resistance that developed in the mutants, we inactivated the penA* alleles of four randomly selected mutants containing Cys69Phe, Arg164His, Glu166Lys, or Ala172Thr substitutions by replacing a region spanning 196 bp in the middle of the coding region with a *tet* ^r^ cassette (see Materials and Methods). All four isolates with inactivated penA* (that are, with ΔpenA*s) lost resistance to ceftazidime (MIC ≤1.75 µg/ml), having an MIC comparable to the level of the wild-type strain with Δ*penA* (i.e. E264ΔP) (Table S1). When a mutant strain with ΔpenA* was provided with an intact copy of the penA* in trans, ceftazidime resistance was restored (Table S1), indicating that penA* was the factor responsible for ceftazidime resistance.

This penA* selection method conducted in its native (wild-type) host simply under a high level of antibiotic pressure without an added mutagen (that is, without an unnaturally elevated mutation rate, which may result in simultaneous multiple mutations), enabled us to investigate the functional adaptability of PenA at the level of single mutations with clear phenotypes. Furthermore, a short incubation time of 16–20 h under selection, that was the same as the normal growth time required for the wild-type strain, ensured that the mutant enzyme sustained a normal growth rate under high antibiotic pressure. In fact, the same growth rate as that of the wild-type was observed with all of the mutants (Fig. S1). Therefore, the set of acquired PenA* enzymes provided information about the extent to which changes in amino acid residues are able to confer high hydrolytic activity against ceftazidime, while maintaining the necessary integrity of the enzyme. In contrast, approaches employing artificial mutagenesis often generate multiple mutations, and in these cases interpreting the contribution from each mutation is not straightforward [4]. Furthermore, many such mutations confer only weak hydrolytic activities and/or cause low enzyme stability, thus making the mutations unsuitable for selection in natural and/or clinical settings. Although clinically occurring mutations that evolve in a patient treated with antibiotics may not have issue of enzyme stability, many of them do not have clear phenotypes associated with substrate spectrum expansion and/or only confer weak β-lactam hydrolyzing activity [4].

### Twelve Amino Acid Positions in PenA were Substituted, each with Distinct Patterns

A total of twelve amino acid positions were repeatedly found substituted in 516 mutated PenAs (Fig. 1a). Ten of the substituted amino acids were located in the omega loop, a structural domain constituting part of the active-site pocket [18]. One of the four conserved domains [19] in class A β-lactamases,^ 166^EXXLN^170^ (numbering following Ambler *et al.* [17]), is located in the omega-loop. Notably, all three conserved residues in this domain were substituted in the set of PenA mutants. The other two substitution positions were located close to, but not in, two other conserved domains, ^70^SXXK^73^ and ^130^SDN^132^. However, no substitution was found near the fourth domain, ^234^KTG^236^ (Fig. 1a).

Each of the twelve positions exhibited one to four different substitutions out of five to seven substitution choices considering all possible single nucleotide changes in the codons, totaling twenty nine substitutions (Fig. 1b). Most (nine of the twelve) positions had more than two types of substitutions, reflecting that the major impact of the substitutions is more caused by which residues are replaced rather than which replaced the original residues. However, the fact that not all possible residues were found in the substitutions at each position (Fig. 1b) suggests that there are specific qualifications required by the substitute residues in each substitution. In this regard, it is notable that positions Asn170 and Ala172 exhibited a preference for a molecular weight (MW) range such that the substitutes were the largest three or four among the candidates, respectively, regardless of the different R-group properties (Fig. 1b). Assuming that the MW roughly correlates with occupying volume by a residue, these substitution patterns suggest that steric factor may play a role in these substitutions. Furthermore, the two substitutes for position Cys69 share an intriguing commonality; both are aromatic residues. In contrast, three positions Leu162, Thr171, and Asp179 had only one-type of substitutions (Fig. 1b). At least at positions Leu162 and Asp179 that had a large number of associated isolates (26 and 54, respectively), the selected substitute residues may be the only qualified ones. Notably, these residues Leu162 and Asp179 are present at the either side of the outer boundary of the omega loop, suggesting that these specific locations may limit diverse substitutions at these positions compared to those inside the omega loop. Although structural data will be needed to understand the specific changes made by each substitution, our data formulated by a large number of mutants clearly unveiled the possible evolutionary paths of the class A β-lactamase during substrate spectrum expansion.

### Each Substitution Exhibited a Distinct MIC Level and Frequency of Occurrence

The Glu166Lys and Leu169Gln substitutions, which occurred in the ^166^EXXLN^170^ domain located in the omega loop, resulted in the highest ceftazidime-hydrolytic activity (Fig. 1a). Interestingly, the Cys69Phe substitution, which occurred outside the omega loop directly upstream of the essential catalytic Ser70, also resulted in one of the highest MICs for ceftazidime. That the Glu166Lys substitution in PenA exhibited high ceftazidime-hydrolyzing activity (Fig. 1ab and Table S1) is intriguing, because no substitution at the position Glu166 has been found in clinical isolates of class A β-lactamases. Glu166 has been reported to play a key role in β-lactam hydrolysis catalyzing the deacylation step after the acylation step catalyzed by Ser70 leading to the formation of an acyl-enzyme intermediate with the β-lactam acylated to the side chain of Ser70 [19]. This deacylation-deficient PenA* with Glu166Lys may produce resistance to ceftazidime by a covalent-trapping mechanism as suggested by a recent study with a deacylation-deficient mutant TEM with a Glu166Arg substitution [20].

Among more than two substitutions at some positions, there were those that better served in terms of the ceftazidime-hydrolytic activity. Most notably, the three positions in the conserved domain ^166^EXXLN^170^, Glu166, Leu169, and Asn170, showed distinct MIC levels depending on substitutions, and those with dissimilar residues appeared to result in high impacts (Fig. 1a). Most notably, the substitution with highly dissimilar Lys (basic) at position Glu166 (acidic) showed a much higher level of MIC compared to the substitutions with nonpolar Gly or Asp, which is also acidic (Fig. 1ab). At position Leu169 (nonpolar), the substitution with nonpolar Pro showed the lowest MIC compared to the substitutions with Gln (polar) or Arg (basic). In addition, two of the three substitutions of Asn170 were with aromatic residues, resulting in higher MICs than the third substitution with Lys (Fig. 1ab). In class A β-lactamases, the terminal amide group of Asn170 has been proposed to play a critical role in the catalysis of β-lactams by positioning a water molecule in the vicinity of the catalytic Ser70 through a hydrogen-bond network [21]. As the Asn170 mutants may not have a normal hydrogen bond network, it remains to be studied how these enzymes maintain their catalytic activity, and further, how the aromatic substitutes result in high hydrolytic activity against ceftazidime.

Our data demonstrate that the MIC levels and the relative frequency in the occurrence of substitutions did not correlate well with each other (Fig. 1b). This low correlation may be because the difference in ceftazidime-hydrolyzing activity levels may not be a critical factor once all of the mutants surpass a threshold-level of hydrolytic activity required for survival. In fact, our growth experiment showed the same growth rate for all mutants, which is comparable to that of the wild-type strain (Fig. S1). It has been known that the intrinsic nucleotide-level mutational biases present in bacteria; G→A and C→T transitions have been found to be the most common non-synonymous mutations [22]. Additionally, a higher prevalence of the transversion A↔T than of A→C or T→G has previously been observed in the *B. mallei* genome [22]. In this regard, it is notable that three of the most frequent nucleotide substitutions at the twelve positions were indeed the transitions G→A (Glu166Lys and Asp179Asn) and C→T (Ala172Val) (Fig. 1b). In addition, at positions Cys69, Arg164, Ala172, and Asp176, where multiple substitutions occurred, the transitions G→A and C→T were highly preferred (Fig. 1b). However, the preference in the transversion A↔T was not observed in the PenA substitutions (Fig. 1b).

**Figure 2:**
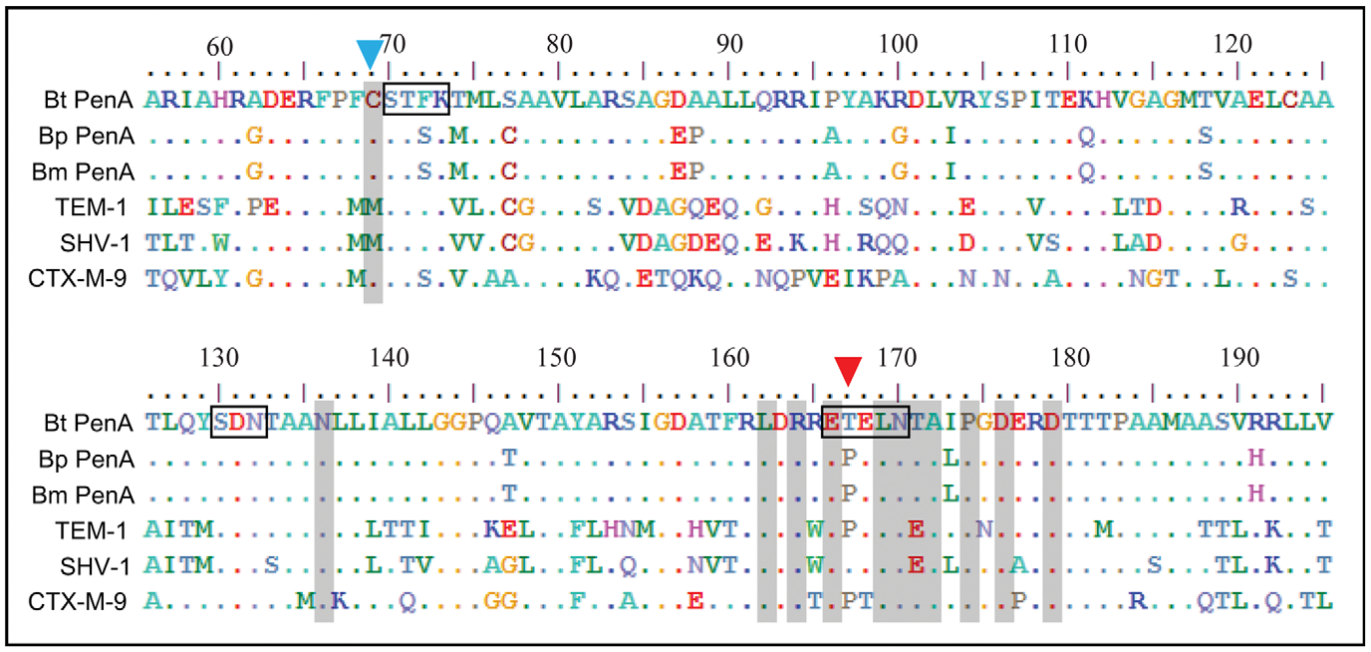
Sequence conservation of the 12 substituted positions in PenA among class A β-lactamases. PenA proteins from *B. thailandensis* (Bt), *B. pseudomallei* (Bp), and *B. mallei* (Bm) and representatives of the three largest families of class A β-lactamases, TEM-1, SHV-1, and CTX-M-9, are aligned. The twelve positions with changes in Bt PenA* are denoted in shades of gray. Positions 69 and 167 have residues in other β-lactamases that differ from those of *B. thailandensis* PenA, which are denoted with blue and red arrowheads, respectively. Three of the four conserved domains in class A β-lactamases, ^70^SXXK^73^, ^130^SDN^132^, and ^166^EXXLN^170^, are denoted by boxes on the Bt PenA sequence.

Nine of the twenty nine substitutions were found in clinical isolates of class A β-lactamases (Fig. 1b), and five of these substitutions (Cys69Tyr [16], [23], Arg164His [24], Arg164Ser [24], Asp179Asn [24], and Leu169Arg [25]) have been verified to cause substrate spectrum expansion in *B. pseudomallei* PenA or TEM- or SHV-type enzymes. Among the twelve substitution positions, only one (Asp179) had additional substitutions that were found in TEM- or SHV-type ESBLs but not obtained in our PenA mutation set (Fig. 1b). As Asp179 could be further substituted, besides by Asn, by Gln in TEM- and by Gly and Ala in SHV-type ESBLs (Fig. 1b), it appears that substitution at position 179 is affected by specific local environment within each β-lactamase. On the other hand, Met is present instead of Cys at position 69 of TEM-1 and SHV-1 (Fig. 2). Therefore, derivatives of these enzymes displayed substitution patterns different from those in PenA (TEM and SHV Tables summarizing variants in each group can be found at http://www.lahey.org/Studies/). Position 171 of TEM-1 and SHV-1 also has a different residue (Glu instead of Thr) compared to PenA (Fig. 2), however no derivatives of the enzymes have been identified at this position. Except for positions 69 and 171, all other ten substituted positions in PenA have the same well-conserved residues as in the other enzymes, including PenA homologs in *B. pseudomallei* and *B. mallei* and the representative enzymes, TEM-1, SHV-1, and CTX-M-9, that do not have activity against ceftazidime [26] (Fig. 2). The sequence conservation at these positions suggests a pivotal role played by these residues, and this in turn suggests that the expansion of the substrate spectrum in class A β-lactamases may follow a common evolutionary trajectory. On the other hand, Pro is present at position 167 in *B. pseudomallei* instead of Thr as in *B. thailandensis* PenA (Fig. 2), and therefore the Pro167Ser mutation found in clinical isolates of *B. pseudomallei* [11] was not obtained in our set of mutants. Although the effect of this mutation has been confirmed in *B. pseudomallei* [23], whether the same affect applies to *B. thailandensis* PenA remains to be verified, as the substitute Ser167 is similar to the original residue Thr167 in *B. thailandensis* PenA.

### The Twelve Amino Acid Positions are Co-localized in the Enzyme and Appear to cause a Perturbation in the Omega Loop

**Figure 3:**
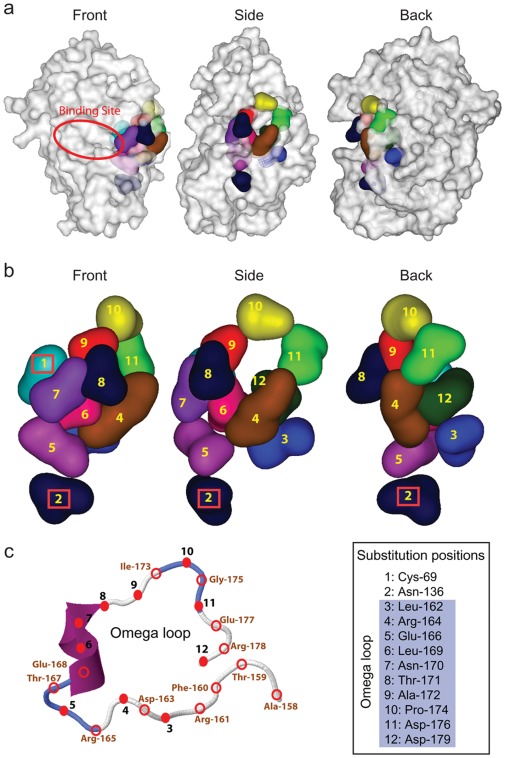
Simulated structure of wild-type PenA. The twelve amino acid positions that are targets for substitution are highlighted. a. Clustering of the twelve amino acid residues in PenA. The front, side, and back views of the simulated structure are shown. Each amino acid residue is color-coded for distinction. The predicted binding site, identified using CASTp [48] by comparing PenA with the binding site of CTX-M-9 interacting with cefoxitin, is denoted with a red closed circular line. b. A closer look at the twelve-residue cluster. The front, side, and back views of the twelve-residues, each numbered from 1 to 12, are shown. Red boxes denote residues Cys69 (1) and Asn136 (2), which are distinct from the rest of the residues because they are not part of the omega loop. C. The omega loop structure with ten substituted residues denoted by red solid circles and the other residues by unfilled circles.

To investigate the structural changes in PenA that correlate with the substrate spectrum expansion to ceftazidime, we conducted modeling analyses using CTX-M-9 covalently linked to cefoxitin (PDBID: 1YXM, 58% of amino acid identity to PenA) [27] as a template. We chose CTX-M-9 for the simulation because of its high homology to PenA and it does not hydrolyze ceftazidime similar to wild-type PenA [26]. Simulated 3D models of PenA showed that the two substituted amino acid positions 69 and 136 co-localized at the active-site pocket area of the enzyme along with the ten positions in the omega loop (Fig. 3a, b, c).

**Figure 4:**
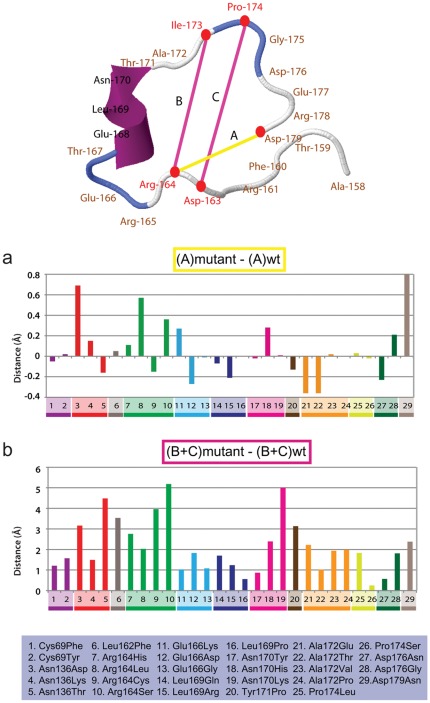
Altered distances in the omega loop in PenA*. The measured distances are denoted A, B, and C in the simulated 3D omega loop structure. a. Alteration in the distance between positions 164 and 179. The distance between positions 164 and 179, where the ionic bond is present (at least in the wild type), of each of the PenA*s is compared to that of the wild-type in a bar graph. b. Altered internal space in the omega loop in PenA*s. The sum of the distances between positions 164–173 and 163–174 in the omega loop is compared to that of the wild-type in the bar graph.

In our simulations, the size and shape of the binding pocket did not show significant correlation with each amino acid substitution. Instead, we observed that the most substitutions of Arg164 or Asp179 (except Arg164Cys), disrupting the stabilizing salt bridge between the two residues that clamp the omega loop structure at both ends [18], resulted in increased distances between positions 164 and 179 (Fig. 4a). This is consistent with previous studies that demonstrated that disruptions of the ionic bond between residues 164 and 179 destabilize the omega loop [19], [28], [29]. Notably, the Asp179Asn mutant, which had a higher MIC than the Arg164 mutants (Fig. 1a), also had a longer 164179 distance in our analysis (Fig. 4a). In addition, PenA*s with Asn136Asp, Glu166Lys, or Asn170His, also showed significantly increased distances between positions 164 and 179, suggesting a possibility that the altered local environment created by some other substitutions may also affect the interaction between Arg164 and Asp179 (Fig. 4a).

Although each of the amino acid substitutions may have caused diverse local disturbances, all twenty nine mutations had the same congruent effect. Specifically, we noted that the space in the omega loop (calculated as the sum of the distances between amino acid residues 163 and 174 and between 164 and 173) increased in all PenA*s, including those with substitutions outside the omega loop, compared to the wild-type PenA (Fig. 4b). We postulate that increased space in the omega loop may accompany increased flexibility of the loop in the mutant enzymes. Then, this flexibility would in turn relieve steric hindrance between the omega loop and the bulky 7β-side chain of ceftazidime, thereby increasing accessibility of ceftazidime to the binding pocket [30], [31].

### PenA*s have Altered Activities for Hydrolyzing Various β-lactam Antibiotics

Our data comparing the MICs of the wild-type strain E264 and the various mutant strains showed that PenA is responsible for the intrinsic moderate insensitivity of the wild-type strain E264 to various classes of β-lactam antibiotics, including amoxicillin, ceftriaxone and cefotaxime (a 3^rd^-generation cephalosporin), and cefepime (a 4^th^-generation cephalosporin) (Table S1).

To investigate the extent to which the structural changes in the PenA*s that were adjusted to ceftazidime affect the enzyme activity towards other β-lactam antibiotics, we measured the MICs for selected β-lactam antibiotics. These antibiotics included the four that the wild-type enzyme was able to hydrolyze and a carbapenem antibiotic (meropenem) and a β-lactamase inhibitor (clavulanic acid with amoxicillin). All PenA*s generally exhibited decreased levels of resistance to the original substrates as observed in many mutants of the class A β-lactamase that acquired activity to 3^rd^ generation cephalosporins [32] (Table S1). The hydrolytic activities of all PenA*s against amoxicillin (MIC range: 3 ∼ 24 µg/ml) were effectively inhibited by clavulanic acid. In addition, resistance to meropenem was not observed in the wild-type or the mutants (Table S1).

### A Second-phase Selection did Not Result in a Mutation in the Coding Region but Rather in the Promoter of *penA*

**Figure 5:**
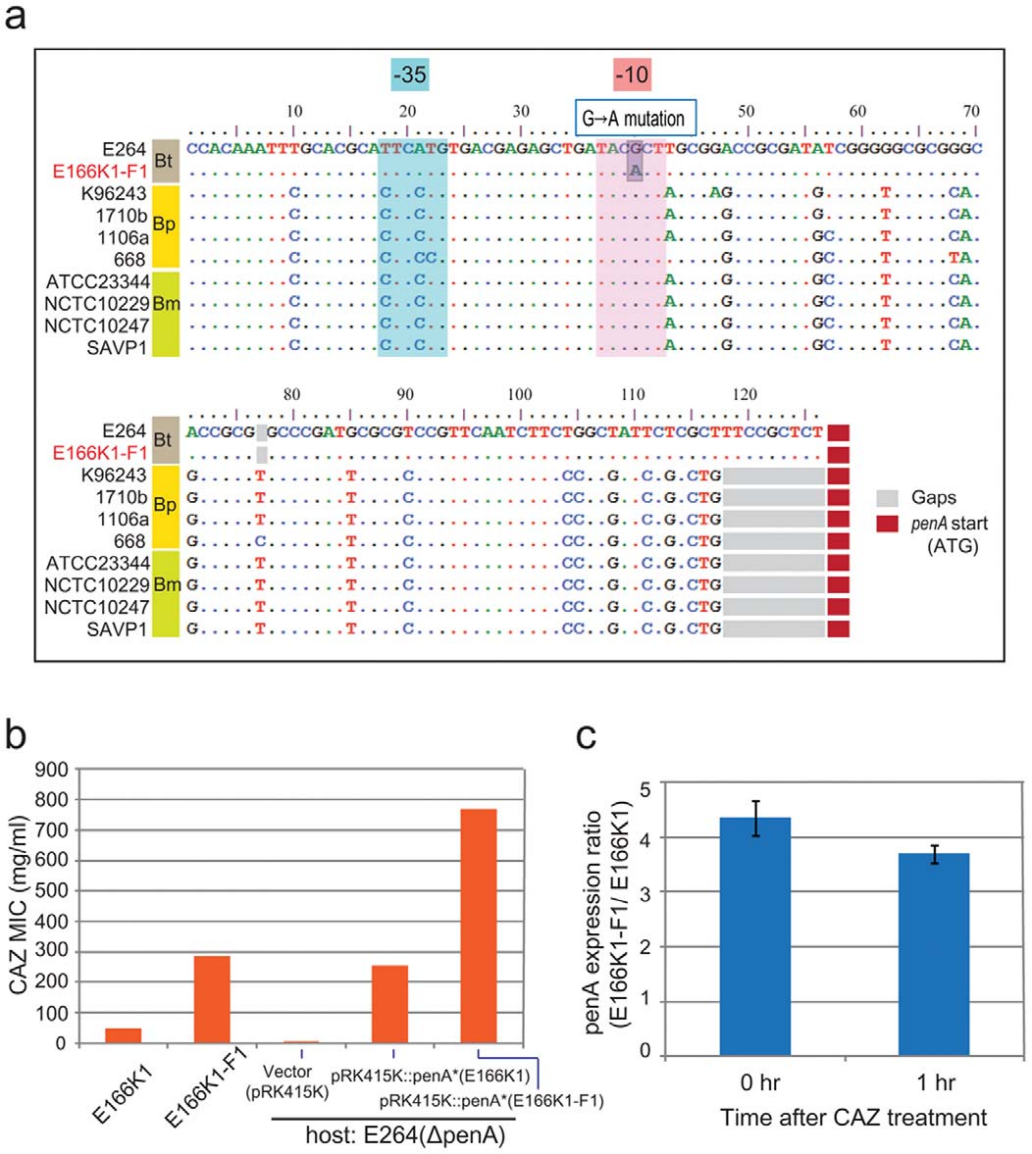
Mutations from the second-round selection with an increased ceftazidime level. a. The G→A point mutation found in the putative promoter region of *penA*. Nucleotide sequences of the wild-type (E264) and the mutant (E166K1-F1) with a point mutation in the putative promoter of *penA* (E166K1-F1) of *B. thailandensis* (Bt) are shown and are aligned with multiple sequences from *B. pseudomallei* (Bp) and *B. mallei* (Bm) strains. The point mutation is denoted with a label and highlighted. The putative -10 and -35 regions are denoted in shades of red and blue, respectively, and putative start codons (ATG) are denoted with red boxes. b. Ceftazidime-hydrolyzing activities of the promoter mutant. The ceftazidime (CAZ) MIC values of a promoter mutant (E166K1-F1) and its parental strain (E166K1) are compared in the bar graph. Vector-carried penA* fragments from E166K1-F1 and E166K1 are also compared in the background of E264(Δ*penA*). c. Comparison of the transcription levels of the promoter mutant (E166K1-F1) and its parental strain (E166K1). The relative expression levels measured by microarrays are shown in the graph.

To investigate whether PenA*s accumulate more mutations under an elevated level of ceftazidime, we conducted a second-level selection with three randomly chosen PenA mutants, containing Cys69Phe, Glu166Lys, or Asp179Asn (see Materials and Methods). The selection process did not result in any additional changes to the coding region, but the same single point mutation, G→A in the region upstream of *penA*, was observed in seven of the eleven second-phase mutants derived from all three mutants (Fig. 5a). Intriguingly, this mutation also has been identified in a clinical isolate of *B. pseudomallei*, and was noted conferring CAZ resistance [16]. The G→A point mutation lies in the putative -10 sequence 5′-TACGCT-3′, changing it to 5′-TACACT-3′, which is closer to the consensus sequence for the -10 sequence in *E. coli*, 5′-TATAAT-3′. One of these mutants, E166K1-F1, derived from the mutant E166K1 with Gln166Lys, was further investigated. This mutant showed a significant increase in hydrolytic activity against ceftazidime compared to the parental strain E166K1 (Fig. 5b). The double mutant penA* fragment from E166K1-F1 carried by pRK415K conferred an approximately three-fold increase in MIC value compared to a similar clone with a fragment from E166K1 in the E264(Δ*penA*) background (Fig. 5b). To determine whether the point mutation resulted in increased penA* expression, we compared the transcriptome profiles of E166K1-F1 and E166K1 obtained before and 1 h after ceftazidime challenge (see Materials and Methods). Intriguingly, the basal level expression of penA* was approximately four-fold higher in E166K1-F1 than in E166K1, but there was no further induction in response to the addition of ceftazidime (Fig. 5c). In contrast, no other genes were differentially expressed as highly as penA* in E166K1-F1 compared with E166K1 (data not shown). In addition to the promoter mutations, there were a smaller number of other isolates (four of the eleven) for which we could not find a mutation in the promoter or in the coding region of penA*. This finding suggests that there may be an additional determinant that contributes to resistance to ceftazidime in *Burkholderia thailandensis*.

### Perspectives

In our mutant selection, a large proportion of the mutations in the bacterium were expected to occur in *penA*; however, the finding that all 516 ceftazidime-resistant isolates that we collected had a mutation in this gene was intriguing. This result strongly suggests that the class A β-lactamase PenA is a predominant determinant for the development of ceftazidime resistance in *B. thailandensis* and perhaps more widely in *Burkholderia* spp. This notion is supported by the fact that most of the validated mutations associated with ceftazidime resistance in *B. pseudomallei* and *B. cepacia* have to date been located in *penA* homologs [9], [10], [11], [23], [33]. In the second-phase mutant selection with an increased antibiotic challenge, we obtained mutations that were not associated with *penA* but were present elsewhere in the genome, demonstrating the presence of other genetic determinants. In clinical *Pseudomonas aeruginosa* isolates, which often lack class A β-lactamase genes, the development of ceftazidime-resistance could be a result of various mechanisms with multiple determinants [34]. There certainly is a possibility that homologs of these *P. aeruginosa* genes, including those coding for class B and D β-lactamases, efflux pumps, and porins, may have mutations associated with ceftazidime resistance.

The levels of ceftazidime resistance in clinical *B. pseudomallei* isolates [35], [36] and in Bcc [37], [38] have been determined to be low. However, the continued use of ceftazidime in clinical settings suggests the potential for the increased emergence of ceftazidime resistance in these bacterial groups. Furthermore, as SHV and CTX-M enzymes are thought to have originated from chromosomal β-lactamases of *Klebsiella pneumoniae* [39] and *Kluyvera* species [6], respectively, *Burkholderia penA* also has the potential to cause a global impact if it becomes mobile.

Substitutions in the omega loop, especially at positions 164, 166, and 179, have been reported to seriously affect the stability of TEM β-lactamases [20], [32]. However, the Met182Thr substitution was shown to restore the stability of these enzymes [20], [32]. As PenA*s with a perturbation in the omega loop produced high ceftazidime-hydrolyzing activity without obvious growth retardation of the host bacteria (Fig. S1), it is intriguing to note that PenA contains a Thr residue at position 182 (Fig. 2). However, it remains to be investigated if the Thr182 in PenA contributes as a global stabilizer for the vast array of substitutions we obtained in the active site area.

Mapping twelve positions and their twenty nine single amino acid substitutions in a class A β-lactamase capable of altering the substrate spectrum of the enzyme is unprecedented. Notably, only seven of these twelve positions and nine of the twenty nine mutations have been reported in clinical isolates of TEM- or SHV-type β-lactamases, or in the PenA homologs of other *Burkholderia* spp., and not all of these have been reported with confirmation in their effects on the substrate spectrum expansion. These data have substantial value, representing a comprehensive repertoire of enzyme adaptability that can occur in clinical settings. These mapped amino acid positions and substitutions with clear ceftazidime resistance phenotypes provide an invaluable resource for the development of preventive strategies and treatments against not only ceftazidime resistance in pathogenic *Burkholderia* species but also in ESBLs in general that share functional mechanisms in their evolution against new drugs using common features of conserved residues.

## Materials and Methods

### Bacterial Cultures

All *Escherichia coli* strains were grown in Luria Bertani (LB) media, and all *B. thailandensis* strains were grown in LB or AB minimal media containing 0.25% glucose (ABG) [40] at 37°C. The concentrations of antibiotics used for *E. coli* were as follows: tetracycline, 10 µg/ml; kanamycin, 50 µg/ml; and ampicillin, 100 µg/ml. For *B. thailandensis*, the concentrations of tetracycline and kanamycin used were 50 µg/ml and 250 µg/ml, respectively.

### Determination of the MIC (Minimal Inhibitory Concentration) Values

The MIC values were measured by the E-test [41], following the manufacturer’s instructions (AB Biodisk, Solna, Sweden). Briefly, each strain was grown on Müller-Hinton agar plates at 37°C for 2 days. For the strains harboring pRK415K-derived plasmids, Müller-Hinton agar supplemented with kanamycin (250 g/ml) was used. Single colonies from the plates were suspended in 1 ml PBS until the turbidity reached 0.5 MacFarland standard. Using sterile cotton swabs, cell suspensions were spread on the Müller-Hinton agar plates, the E-test strips were placed, and the plates were incubated at 37°C for 16∼18 h. The lowest concentrations at the E-test strips where no visible growths were observed were recorded as the MICs. Average values were calculated from triplicate experiments.

The agar dilution method was conducted as described by Wiegand *et al.* [42], except that LB agar was used instead of Mueller Hinton agar. Briefly, a single colony of each strain grown on ABG minimal medium agar was used to inoculate 2 ml of LB broth, and the culture was incubated overnight with shaking (250 rpm) at 37°C. The overnight cultures were serially diluted with fresh LB and dispensed into the wells of a 96-well microtiter plate. Using a multi-channel micropipette, 1 µl of the diluted bacterial suspensions was placed on each LB agar plate containing 0.5 to 8 µg/ml of ceftazidime and incubated at 37°C for 16 to 20 h. The lowest concentration of antibiotic such that there was no visible bacterial growth was observed in the spot containing approximately 10^4^ CFU (colony forming unit), which was recorded as the MIC. The number of CFUs in serially diluted bacterial suspensions was determined by spreading 100 µl of the appropriately diluted bacterial suspensions on LB agar plates, followed by incubating the plates for 24 h at 37°C, and counting the viable cells. The average values were calculated from triplicate experiments.

### Isolation of Mutants Resistant to Ceftazidime

A single colony of the wild-type *B. thailandensis* strain E264 was grown overnight in 2 ml of LB broth at 37°C with shaking (250 rpm). The overnight culture was pelleted by centrifugation (2,600 X g) for 5 min at 4°C, and the cells were resuspended in fresh LB broth to approximately 10^9^ CFU/ml. Cell suspensions (100 µl) were spread on LB agar plates containing 5 µg/ml (slightly lower than three times the wild-type MIC) ceftazidime, which were subsequently incubated for 48 h at 37°C until ceftazidime resistant mutants formed visible colonies. We picked two to three colonies from each selection plate to collect up to 516 isolates for this study. A second-round selection was conducted with an elevated concentration of ceftazidime (96 µg/ml) with selected ceftazidime-resistant mutants.

### Localization of the Mutations in *penA*

To map the mutations in *penA* (BTH_II1450 in the strain E264 genome), we first set out to sequence the gene. Genomic DNA of each ceftazidime-resistant mutant was purified using a Wizard Genomic DNA Purification Kit (Promega, Madison, USA) and was used as the template for PCR amplification of *penA* and short flanking regions for a 1,386 bp amplicon (271 bp upstream from the start codon of the gene to 230 bp downstream of the stop codon). PCR reactions were conducted in a 50 µl reaction mixture containing 2.5 U of HotStar HiFidelity polymerase (Qiagen, Hilden, Germany), 50 pmol of the primers penA-F (5′-CGTCAATCCGATGCAGTACC-3′) and penA-R (5′-GCCGTTATCGCACCTTTATC-3′), 100 ng template DNA, 10 µl of 5X Q solution, and 10 µl of 5 X HotStar HiFidelity buffer. The reaction consisted of the following three steps: initial enzyme activating step (95°C for 5 min), amplification step (35 cycles of 94°C for 15 sec, 61°C for 1 min, and 72°C for 1.4 min), and final extension step (72°C for 10 min). Gel-purified 1.4 kb PCR products were sequenced using a 3730XL DNA analyzer (Applied Biosystems, Foster City, CA, USA) in both directions using primers penA-F and penA-R.

### Construction of *penA-*null Mutants

The wild-type and mutated *penA* genes were disrupted as follows. First, the wild-type *penA* was PCR-amplified, using the primer pairs penA-KF (5′-ATATATGGTACCCGTCAATCCGATGCAGTACC-3′) and penA-KR (5′-ATATATGGTACCGCCGTTATCGCACCTTTATC-3′), containing a *Kpn*I recognition site (underlined) at the end. Then the 1.4 kb PCR product was digested with *Kpn*I and ligated into pUC19, which was digested with the same enzyme. The resulting plasmid was double-digested with *Xho*I and *Pfl*FI to remove an internal region (position 124 bp to 319 bp) of *penA,* which is 885 bp long, blunt ended with T4 DNA polymerase (NEB, Ipswich, MA, USA), and were inserted with a tetracycline-resistance (*tet* ^r^) cassette by ligation. The *tet* ^r^ cassette was previously amplified from pRK415K [43], using HotStar HiFidelity polymerase (Qiagen, Hilden, Germany) and primers tetR-F (5′-ATATATCTCGAGGTGAGGCTTGGACGCTAGG-3′) and tetR-R (5′-ATATTTCTCGAGCTTGGATCAGACGCTGAGTG-3′), containing a *Xho*I recognition site (underlined), *Xho*I-digested, and blunt-ended. The construct was transferred into *B. thailandensis* strains employing a modified method of natural transformation [44]. Briefly, 3 ml of a defined medium (DM) prepared as described by Thongdee *et al*. [44] was inoculated with a single colony freshly grown on LB agar and was incubated overnight with shaking at 250 rpm at 37°C. The overnight culture of 200 µl was diluted with 10 ml of fresh preheated DM and the culture was grown with shaking (250 rpm) at 37°C to an OD_600_ of approximately 0.5. The culture was then concentrated 20-fold in 500 µl fresh DM and 50 µl aliquots of the concentrated cells were mixed with 0.5 µg of plasmid DNA. The mixture was incubated for 30 min on ice and 2 ml DM preheated to 37°C was added and the mixture was incubated overnight at 250 rpm at 37°C. After washing the culture with 1 ml of fresh DM and resuspending the pellet in 250 µl of fresh DM, 100 µl of the cell suspension was plated on ABG medium containing 50 µg/ml tetracycline and incubated at 37°C for 48 hrs to select for *tet* ^r^ cassette-containing constructs. An obtained *penA* null mutant was verified by PCR using a primer pair penA_LF (5′-AACAGATCGCCGAGATGG-3′) and penA_LR (5′-GCGAACGTTGCCCGATAC-3′) that hybridize to the genomic regions outside the DNA sequence used for mutant construction.

### Complementation of the *penA* Mutations

*Kpn*I-treated PCR products of penA* (*penA* with a single nucleotide mutation), which was amplified using the primers penA-KF and penA-KR as described above, was ligated with *Kpn*I-treated pRK415K. This plasmid was transferred into the *E. coli* strain S17-1 [45] by using a conventional transformation method [46]. The transformed S17-1 strain was conjugated with a *B. thailandensis penA-*null mutant on ABG agar plates containing 250 µg/ml kanamycin, and the plates were incubated at 37°C for 2 days to select for transconjugants. A successful conjugation was confirmed by purifying the plasmid from transconjugants and examining the characteristic restriction patterns of the plasmid.

### Modeling Analysis

Because the structure of PenA is not known, the structures (for both mutants and wild-type) were predicted by homology modeling using CTX-M-9 (PDBID: 1YXM, 58% of identity) [27] as a template with SYBYL-X (Tripos Inc., St. Louis, MO, USA). With the predicted structures, molecular dynamic simulations were conducted using OpenMM Zephyr 2.0.3 [47] for 500 ps (0.002 ps/step) at 303.15 K with the Amber03 force field to release any structural constraints originating from the template. Then, energy minimization was performed using SYBYL -X (Tripos Inc., St. Louis, MO, USA) with a Tripos force field until the energy gradient reached 0.001 kcal/(mol•A).

### Gene Expression Study

Gene expression was assayed using microarray technology. The bacterial culture was incubated in LB media until the OD_600_ reached 0.5, then 2 ml of the culture was taken and mixed with 4 ml of RNAprotect bacteria reagent (Qiagen, Hilden, Germany) to prevent alterations in the transcriptome. For cultures that were induced by ceftazidime, a sub-MIC level of ceftazidime (24 µg/ml) was added, the samples were withdrawn after 1 hr, and were immediately mixed with two volumes of RNAprotect bacteria reagent. Total RNA isolation and labeling with Cy3 and Cy5 fluorescence dyes were conducted following PFGRC SOP (http://pfgrc.jcvi.org/index.php/microarray/protocols.html).

Labeled samples were hybridized to the *B. thailandensis* whole genome microarray (MYcroarray, Ann arbor, MN, USA) in a hybridization chamber in a 42°C water bath for 16 to 20 hrs. Microarray slides were washed twice with 1 x SSPE for 3 min and then with 0.25 x SSPE for 30 sec, and were dried immediately by spinning in a micro-slide centrifuge for 1 min. Hybridized slides were scanned using a GenePix 4000B microarray scanner (Molecular Devices, LLC, Sunnyvale, CA, USA). The scanned images were analyzed using TIGR SPOTFINDER (http://www.tm4.org) to obtain relative transcript levels. LOWESS (locally weighted scatterplot smoothing) normalization of data was executed using the software tool MIDAS (http://www.tm4.org). The resulting data were visualized and further explored by using TMEV (http://www.jcvi.org/cms/research/software).

## Supporting Information

Figure S1 (PDF)

Table S1 (DOCX)
