# Supplementary material for: Twelve Positions in a β-Lactamase That Can Expand Its Substrate Spectrum with a Single Amino Acid Substitution
Source: PLoS One. 2012 May 22;7(5):e37585. doi: 10.1371/journal.pone.0037585 (PMC3358254; doi:10.1371/journal.pone.0037585)
Supplement: Figure S1 — (PDF) [file pone.0037585.s001.pdf]

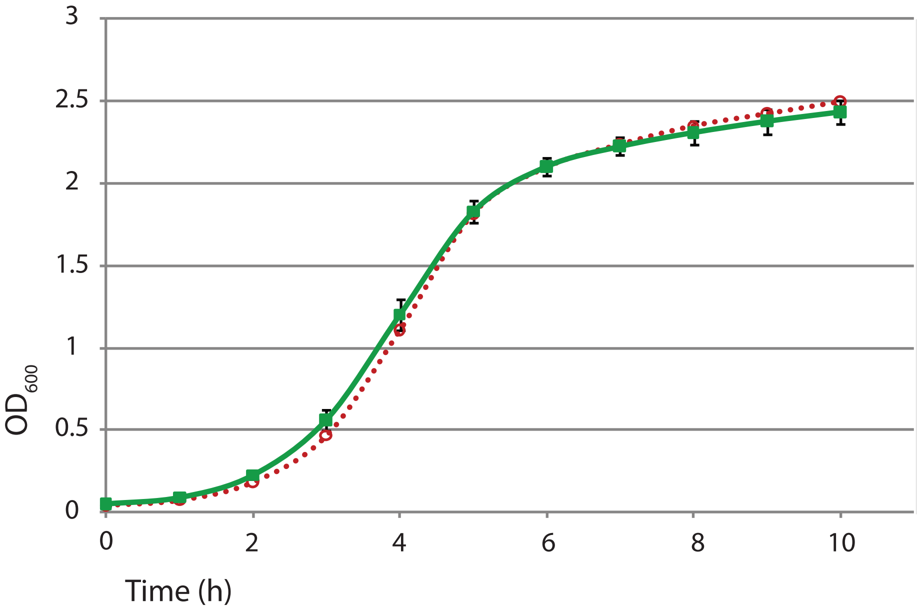

Figure S1. Growth curves of *Burkholderia thailandensis* strains. The average growth curve (green solid line) of the ceftazidime-resistant strains, representing the twenty nine substitutions in the presence of 5 µg/ml ceftazidime, is shown with error bars. The growth curve of the wild-type strain (red dotted line) in the absence of the antibiotic is also shown.
