# Supplementary material for: Twelve Positions in a β-Lactamase That Can Expand Its Substrate Spectrum with a Single Amino Acid Substitution
Source: PLoS One. 2012 May 22;7(5):e37585. doi: 10.1371/journal.pone.0037585 (PMC3358254; doi:10.1371/journal.pone.0037585)
Supplement: Table S1 — (DOCX) [file pone.0037585.s002.docx]

| Table S1. MICs for various β-lactams with *B. thailandensis* strains | | | | | | | | |
| --- | --- | --- | --- | --- | --- | --- | --- | --- |
| Strain | *penA* allele*^a^* | MIC (μg/ml)*^b,c^* | | | | | | |
|  |  | AMX | AMX/CLA | CAZ | CTXM | CRX | CEF | MER |
| **Wild type** | | | | | | | | |
| E264 | *penA* | 36 | 6 | 1.75 | 6 | 16 | 18 | 0.75 |
|  | | | | | | | | |
| ***penA*- or *penA*****^d^***-null mutant strains** | | | | | | | | |
| E264ΔP | *penA* | 4 | 1.5 | 0.75 | 1 | 0.5 | 3 | 0.75 |
| C69FΔP | *penA**(C69F) | 3 | 2 | 0.75 | 1 | 0.25 | 3 | 0.5 |
| R164HΔP | *penA**(R164H) | 4 | 2 | 0.75 | 1 | 0.38 | 3 | 0.5 |
| E166KΔP | *penA**(E166K) | 4 | 2 | 0.75 | 1 | 0.38 | 2 | 0.5 |
| A172TΔP | *penA**(A172T) | 4 | 2 | 1 | 1 | 0.38 | 3 | 0.75 |
|  | | | | | | | | |
| ***penA*-null mutants complemented by *penA* or *penA** carried by a vector pRK415K** | | | | | | | | |
| E264ΔP-C | pRK415K::*penA* | >256 | 48 | 2 | >32 | >32 | 64 | 0.75 |
| C69FΔP-C | pRK415K::*penA**(C69F) | 48 | 1 | >256 | >32 | 24 | 48 | 1 |
| R164HΔP-C | pRK415K::*penA**(R164H) | >256 | 4 | 192 | >32 | >32 | 64 | 0.75 |
| E166KΔP-C | pRK415K::*penA**(E166K) | 32 | 1.5 | >256 | >32 | >32 | 24 | 1 |
| A172TΔP-C | pRK415K::*penA**(A172T) | >256 | 2 | >256 | >32 | >32 | 64 | 1 |
|  | | | | | | | | |
| **Twenty nine mutants with *penA**** | | | | | | | | |
| C69F1 | C69F | 6 | 1.5 | 56 | 3.5 | 3 | 12 | 0.5 |
| C69Y1 | C69Y | 4 | 1.5 | 36 | 3 | 2 | 8 | 0.75 |
| N136D1 | N136D | 8 | 1.5 | 12 | 5 | 4 | 12 | 0.5 |
| N136K1 | N136K | 6 | 2 | 12 | 3 | 3 | 4 | 0.75 |
| N136T1 | N136T | 12 | 1 | 10 | 1.75 | 1.5 | 4 | 0.38 |
| L162F1 | L162F | 16 | 1.5 | 12 | 2.5 | 2 | 6 | 0.38 |
| R164C1 | R164C | 12 | 1.5 | 24 | 3 | 1.5 | 6 | 0.75 |
| R164H1 | R164H | 24 | 2 | 28 | 5 | 4 | 12 | 0.5 |
| R164L1 | R164L | 4 | 1.5 | 28 | 3 | 1.5 | 6 | 0.5 |
| R164S1 | R164S | 12 | 1.5 | 24 | 3.5 | 8 | 12 | 0.38 |
| E166G1 | E166G | 4 | 2 | 12 | 1.5 | 1.5 | 4 | 0.75 |
| E166K1 | E166K | 4 | 2 | 80 | 3.5 | 3 | 8 | 0.5 |
| E166D1 | E166D | 4 | 2 | 16 | 2 | 2 | 4 | 0.75 |
| L169R1 | L169R | 3 | 1.5 | 48 | 3.5 | 2 | 8 | 0.38 |
| L169Q1 | L169Q | 3 | 1.5 | 72 | 3.5 | 4 | 8 | 0.38 |
| L169P1 | L169P | 3 | 1.5 | 24 | 3 | 2 | 4 | 0.5 |
| N170H1 | N170H | 3 | 2 | 24 | 1.75 | 1.5 | 6 | 0.5 |
| N170Y1 | N170Y | 3 | 2 | 32 | 3.5 | 3 | 8 | 0.75 |
| N170K1 | N170K | 6 | 1.5 | 6 | 1 | 2 | 3 | 0.38 |
| T171P1 | T171P | 6 | 1.5 | 16 | 3 | 1.5 | 4 | 0.5 |
| A172T1 | A172T | 16 | 2 | 28 | 6 | 6 | 16 | 0.5 |
| A172V1 | A172V | 16 | 2 | 28 | 15 | 6 | 12 | 0.5 |
| A172E1 | A172E | 6 | 1.5 | 32 | 2 | 1 | 8 | 0.5 |
| A172P1 | A172P | 16 | 1.5 | 24 | 5 | 4 | 8 | 0.5 |
| P174L1 | P174L | 16 | 3 | 9 | 3 | 3 | 8 | 0.38 |
| P174S1 | P174S | 24 | 3 | 8 | 3.5 | 6 | 8 | 0.5 |
| D176N1 | D176N | 16 | 2 | 12 | 3 | 2 | 6 | 0.75 |
| D176G1 | D176G | 12 | 1.5 | 11 | 2.5 | 1.5 | 3 | 0.38 |
| D179N1 | D179N | 6 | 1.5 | 48 | 3.5 | 2 | 8 | 0.5 |

*^a^*Amino acid residues are denoted by single-letter codes and are numbered according to Ambler *et. al*. [[17](#_ENREF_17)].

*^b^*MICs were measured by the E-test.

*^c^*Abbreviations: AMX, amoxicillin; AMC, amoxicillin/clavulanic acid; CAZ, ceftazidime; CTXM, cefotaxime; CRX, ceftriaxone; CEF, cefepime; MER, meropenem; Tc^R^, tetracycline resistance cassette.

*^d^penA** represents a *penA* allele with a single nucleotide substitution in the coding region.
